# The Curious Relation between Theory of Mind and Sharing in Preschool Age Children

**DOI:** 10.1371/journal.pone.0117947

**Published:** 2015-02-06

**Authors:** Jason M. Cowell, Anya Samek, John List, Jean Decety

**Affiliations:** 1 Department of Psychology, University of Chicago, Chicago, Illinois, United States of America; 2 Department of Consumer Science, University of Wisconsin-Madison, Madison, Wisconsin, United States of America; 3 Department of Economics, University of Chicago, Chicago, Illinois, United States of America; National University of Singapore, SINGAPORE

## Abstract

Young children have long been known to act selfishly and gradually appear to become more generous across middle childhood. While this apparent change has been well documented, the underlying mechanisms supporting this remain unclear. The current study examined the role of early theory of mind and executive functioning in facilitating sharing in a large sample (N = 98) of preschoolers. Results reveal a curious relation between early false-belief understanding and sharing behavior. Contrary to many commonsense notions and predominant theories, competence in this ability is actually related to less sharing. Thus, the relation between developing theory of mind and sharing may not be as straightforward as it seems in preschool age children. It is precisely the children who can engage in theory of mind that decide to share less with others.

## Introduction

As a social species, humans routinely engage in acts to benefit others, occasionally at cost to themselves [1,2]. This propensity can be observed very early in development. Indeed, infants appear sensitive to unequal distributions of resources[3], and young children can act prosocially [4]. Preschoolers will share some resources with others [5], and by late childhood (7–8 years of age), children’s sharing approaches equality in distribution [6], though the nature of equality is still disputed [7]. Regardless, in cross-sectional studies of expressed sharing behavior, children in preschool tend to share less than a third of their resources and by late childhood share nearly half [8].

However, while there is considerable change in expressed sharing behavior between infancy and late childhood, it is less clear what mechanisms guide this increase in generosity. In particular, several general cognitive capacities have been proposed to facilitate increased sharing in young children including theory of mind, perspective-taking, and executive function [9–11].

In the case of executive functioning, young children’s apparent selfishness in sharing resources is thought to be an inability to inhibit their own desire for resources. Executive functioning (EF) emerges early in infancy and continues to develop well into adolescence, and parallels the development of the prefrontal cortex [12]. The development of the prefrontal cortex is thought to play an important role in the maturation of higher cognitive abilities including sharing. As individuals develop greater cognitive flexibility, inhibitory control, and working memory, it has been hypothesized that they will better be able to regulate their own desires and engage in prosocial behavior [13]. Surprisingly, empirical results from two recent studies measuring aspects of executive functioning and distributive justice behavior in children have found little evidence for a straight-forward relation between developing EF and sharing behaviors. In the first, 4-to-6 year old children’s inhibitory control (Day/Night stroop task), working memory (Eight boxes task), and cognitive flexibility (Dimensional Change Card Sort task) were assessed in conjunction with a children’s dictator game. In this study, only children’s inhibitory control was related to the number of candies donated (*r* ^2^ = .1), but this relation did not hold when analyses were restricted to only children who had shared any resources [14]. In the second study, inhibitory control as measured by the Day/Night Stroop task was not found to account for the difference between what children said they should share and what they actually shared, but did predict the number of stickers shared (*r* = .26). However, in the same study another measure of inhibitory control (the Bear-Dragon task) was not predictive of sharing behaviors [10].

Previous studies of distributive justice in children have used one of three different types of paradigms, ultimatum/bargaining games [9], forced-choice sharing games with a known confederate [15,16], and dictator games [8,17]. Ultimatum games are thought to measure children’s developing sharing abilities in conjunction with social bargaining. In these tasks, children are required to make an “offer” of how many rewards they want to keep and how many rewards the other child will receive. However, the other child has the ability to reject this offer, resulting in both children receiving nothing. Forced-choice sharing paradigms provide two options to the child, either share with another individual (usually a known classmate) or keep the resources. These games do not allow for children to decide *how* many rewards to share, only whether to share or not. Contrastingly, dictator games with anonymous receivers provide a relatively unbiased assessment of early generosity. In dictator games, children are given a number of rewards (usually 6 or 10) and the option to share none, some, or all of the rewards with another child, who they do not necessarily know (the identity of the other child is not revealed) and who they have no expectation to interact with in the future. As all of these paradigms measure aspects of distributive justice, but require arguably different abilities in the child, the influence of developing perspective-taking and executive function on performance in each will inevitably vary.

For the development of perspective-taking/theory of mind, selfishness is thought to be an early developmental state. By middle childhood, others’ perspectives are integrated into considerations. As children begin to take others’ perspectives, they will recognize others’ desires for resources as well, and subsequently, will be motivated to engage in sharing with a confederate. One study of children’s distributive justice in a bargaining game found that preschoolers with theory of mind abilities tended to offer more resources than those with less mature theory of mind [9]. These findings suggest that taking the perspective of another is valuable in guessing what type of sharing offer the other will accept. Indeed, preliminary relations between perspective-taking and distributive justice indicate that children’s theory of mind development should coincide with greater expression of altruism or sharing. However, to our knowledge, individual differences in early false-belief/theory of mind abilities have not yet been explicitly linked to sharing behaviors in children, particularly sharing behaviors that are not due to social pressure, as is the case in ultimatum/bargaining games.

It should be noted that in most cases, sharing will not necessarily be reciprocated and the expectation of reciprocation is not present [6,16]. Without consequence for resource hoarding (not sharing), children with greater perspective-taking abilities may actually recognize an opportunity for strategic gain at no cost to the self. In this case, children with theory of mind are more selective in their sharing and, where no consequences to selfishness are present and cooperation is not an expectation, will share less with an unknown “other” [18].

Taken together, while generosity develops substantially between infancy and late childhood, the underlying mechanisms of this change are unclear and the directionality of these relations is ambiguous. The current study was designed to examine the early expression of sharing behaviors in a large sample of preschoolers by investigating the roles of theory of mind and executive functioning. Consistent with some recent literature on executive functioning and sharing behavior, it was predicted that executive functioning would be related to explicit sharing (dictator game performance); that greater executive functioning abilities, particularly inhibitory control, would be related to increased generosity in the dictator game.

Additionally, as these children were at the optimal age for variance on false-belief understanding (i.e., 3–5 years), rather than testing relative abilities of theory of mind and their relation to expressive bargaining and sharing [9], in the present study, children could actually be divided into one group that could pass false-locations tasks and one group that could not. The present study directly compared the competing hypotheses that theory of mind abilities in children could either facilitate 1) increased sharing with a peer or 2) decreased sharing with a peer.

## Materials and Methods

### Participants

In the present study, 3 to 5-year-old children (*N* = 98, *M* age = 51.69 months, *SD* = 6.49, *n* = 53 female) were recruited from an ongoing preschool project in a large Midwestern city. Participants were of representative socioeconomic status (primarily low SES) for the greater Chicago area and of diverse races (13.6% Caucasian, 50.5% African American, 35.8% Hispanic).

### Procedure

All children were tested during the school day in a series of assessments. As part of a larger study, executive functioning measures were collected from the preschoolers one month following the collection of theory of mind and dictator game measures. In the theory of mind and dictator assessment session, all children first participated in the standard false-belief task [19], then completed the child modified dictator game [8]. Written informed consent was obtained from all parents, and verbal assent was given by all children in line with ethical guidelines for testing children. All these procedures, including consent from parents and children were approved by the University of Chicago Institutional Review Board.

### Measures

**Theory of Mind**. Theory of mind (ToM) was assessed using the puppet false-belief change location task. In this task, children are shown one puppet, Sally/Steve (gender matched to child), who hides a ball in one of several hiding locations. Sally/Steve leaves the room and another puppet, Anne/Adam, moves the ball from the original hiding location to an alternate hiding location. Sally/Steve then returns to the room and children are asked “Where will Sally/Steve look for the ball?” Children’s theory of mind accuracy is assessed based on whether they point to the original hiding location (the correct answer) *versus* an alternate hiding location (the incorrect answer).

**Dictator Game**. Consistent with previous studies using the dictator game with children, stickers were used [8]. In this version of the dictator game, children were given six stickers and told that “these stickers were now theirs.” They were then asked, “do you like these stickers?” If they did not like a sticker, it was replaced with a new one. They were then told about another child in the educational program that would not be able to play the game and get stickers, but they could give some of their stickers to the other child if they wanted to. Children were asked a series of rule checks to ensure understanding of the task and the directions including asking the child to point to the bag they would put stickers in to take home and the bag that would go to another boy or girl (gender matched) that would not get to play this game. If the child incorrectly identified the bags, the instructions were repeated and the child was asked again. To avoid experimenter bias, and consistent with previous developmental studies using the dictator game, the experimenter turned around during decision-making. Children’s sharing was measured by the number of stickers out of 6 they chose to give to the “other child.”

All executive functioning measures were taken from a nationally validated battery of EF measures used in the Family Life Project, a large scale study of child development (see [20] for a full description of the tasks). For all three executive functioning tasks, the outcome variable of interest was percent accuracy.

**Spatial Conflict Arrows (SCA; [20])**. The SCA is a task that is intended to assess inhibitory control. In the task, children receive a response card with two buttons, one on the left and one on the right. The child is instructed to press the button that matches the direction an arrow is pointing. The task begins with arrows presented centrally in a flipbook, then the arrows appear laterally on the page, matching the side of the target (congruent trials, e.g., the arrow is on the left side of the book and points left). The final 16 trials consist of incongruent trials (e.g., the arrow points left, but is on the right side of the flipbook).

**Something’s the Same Game (STS; [20])**. The STS is a test of attentional shifting/flexibility. In this game, children are shown pages with pictures on them that vary in size, color, and type. The child is asked to identify pairs of pictures that are the same in size, color, or likeness. Once they have identified two objects that match on one dimension, they are told to match two other objects/pictures on another dimension.

**Working Memory Span [20]**. In this task children are shown a drawing of an animal and a colored dot, both located in a house. The child is told to name the color and the animal. The experimenter then flips to a blank house and asks the child “what animal lived in the house?” The task progresses with one, two, three, and four house trials.

## Results

Children shared on average 2.54 candies out of a possible 6 candies (skewness = -0.023, kurtosis = 0.381). To test for potential gender or race effects, a 2 (gender) x 3 (race) Analysis of Variance (ANOVA) with dictator game performance (amount shared) as the dependent variable was conducted. Gender differences in sharing were not significant (*F* (1, 94) = 0.730, *n*.*s*., η^2^ = 0.000), nor were differences by race (*F* (2, 93) = 0.693, *n*.*s*., η^2^ = 0.010). For a breakdown of age, race, and gender distributions of resources, see Table 1. Age-related changes in sharing were also not significant (*r* = .137, *n*.*s*.).

**Table 1:** Descriptives of Sharing in Dictator by race and gender.

Race	Gender	Sharing Mean (SD)	N
African-American	Male	2.58 (1.74)	19
	Female	2.21 (1.29)	29
Hispanic	Male	2.60 (1.60)	15
	Female	2.84 (1.50)	19
Caucasian	Male	3.00 (1.41)	8
	Female	2.20 (1.30)	5

To examine the relation between subcomponents of executive functioning and the amount of sharing, a simple linear regression was performed entering the three components (inhibitory control, attentional flexibility, and working memory) and age in months as predictor variables and amount shared as a dependent variable. This model was not a significant predictor of sharing behavior in children (*R* ^2^ = -0.003, F (4, 84) = .926, *n*.*s*). Moreover, consistent with hypotheses, none of the standardized beta coefficients for executive function measures were significant predictors of sharing including inhibition (*β* = 0.103, *n*.*s*.), attentional flexibility (*β* = -0.120, *n*.*s*.), and working memory (*β* = -0.005, *n*.*s*.).

**Fig 1:**
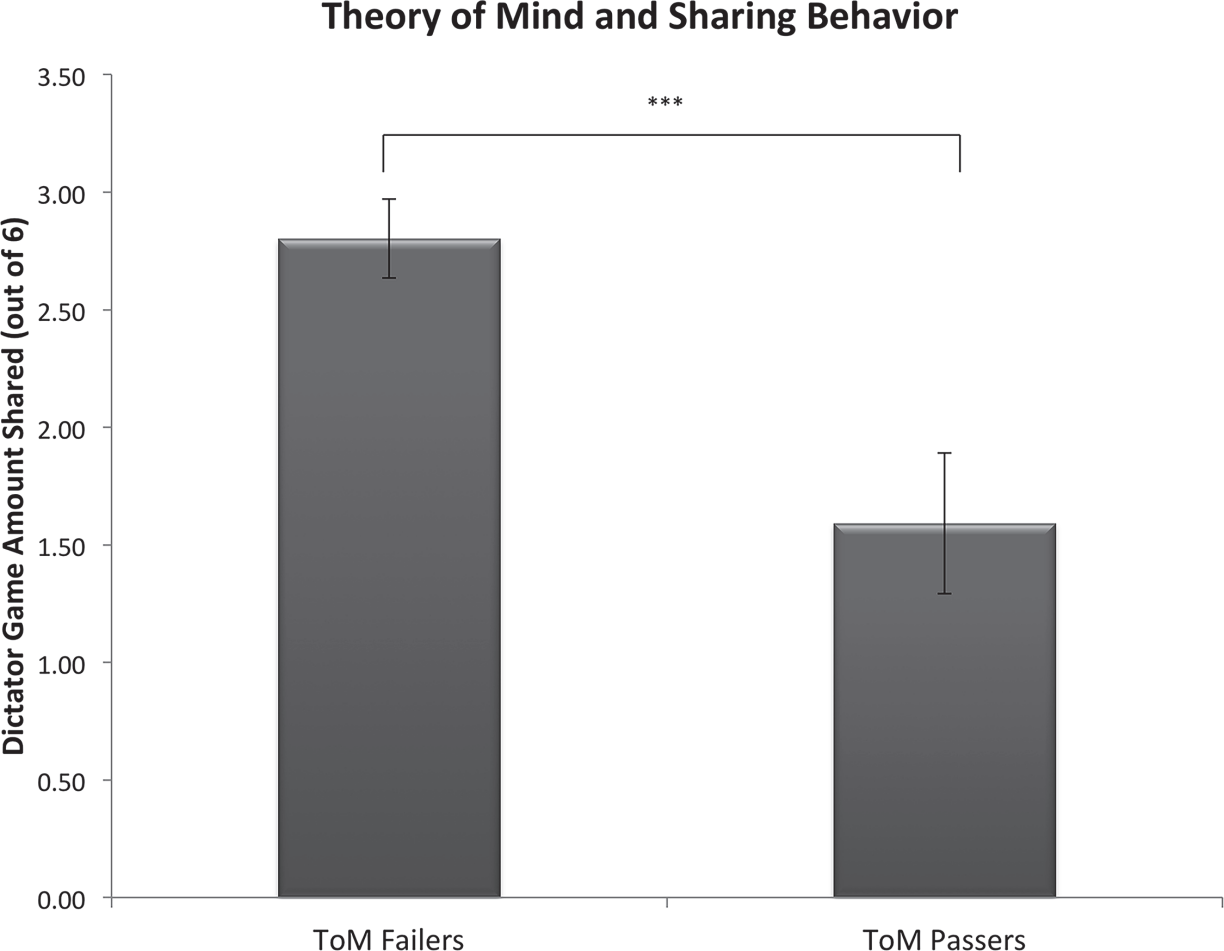
Sharing in Dictator by Theory of Mind Performance. Children who fail a false belief task share significantly greater resources than children who pass a false belief task. *** denotes *p* < .001.

Finally, sharing propensity in the dictator game significantly differed based on false-belief performance (passing/failing), (*t* (91) = 3.603, *p* < .001). Children who passed a test of theory of mind shared an average of 1.59 stickers (out of 6), whereas those who failed shared an average of 2.8 stickers (see Fig. 1). Additionally, sharing behavior differed by false-belief abilities even after using age in months as a covariate (*F* (1, 92) = 15.09, *p* < .001, η^2^ = .121).

## Discussion

To investigate the extent to which changes in generosity are influenced by underlying domain-general cognitive mechanisms, executive functioning and theory of mind were assessed in conjunction with a behavioral economics sharing game in a large sample of preschool age children. Consistent with our hypotheses, theory of mind abilities (as measured by the false-location task) were related to generosity. Surprisingly, children who passed a standard false-belief paradigm shared significantly less resources with their peers than did children who failed a standard false-belief, even after accounting for age-related differences. Importantly, this difference was quite large in size (η^2^ = .121). Contrary to our hypotheses, but consistent with some previous findings [10], children’s selfishness in resource distribution was not significantly related to aspects of executive functioning. Furthermore, no relations between EF and generosity even trended toward significance (all *p’s* > .1).

In general, prosocial behavior is an umbrella concept that encompasses actions that assist another person such as spontaneous helping, sharing, and comforting or empathic helping [21,22]. Importantly, each of these prosocial behaviors requires distinct emotional, social-cognitive, self-regulatory, motivational, and other psychological constituents [23]. The re-emphasis on the study of prosocial behavior in recent years points to substantial development in fairness sensitivity and sharing behavior. Infants and young children appear to be sensitive to unequal distributions of resources [24], however, children as old as 5 years seem unable to act on this knowledge [6]. Previously, this dissociation has been theorized as a developing mental model of fairness, which is thought to be grounded in other-oriented perspective taking and executive functioning, wherein children’s notions of equality change across early and middle childhood based on the development of these abilities [25].

The results from our study provide preliminary evidence that domain-general capacities partially underlie observed changes in the development of generosity across childhood and reflect a curious relation between early perspective-taking and sharing behaviors. Interestingly, perspective-taking abilities in preschool age children were related to sharing, but not in the direction that predominant theories would predict. Those children who engaged in perspective-taking actually shared less with peers than those who did not. These findings may be interpreted in line with a growing body of literature indicating that children begin to see resource distribution as strategic and competitive and are increasingly selective with whom they will share resources with across development [16]. Those who can take another’s perspective may actually understand that there are no consequences for engaging in resource hoarding and act on this knowledge. In the present study, there was no expectation for reciprocity, a motivation that has been argued to heavily influence expressions of sharing [6,26]. In previous work, older children with greater perspective-taking abilities offered more resources in a bargaining game [9]. The results from that latter study keep with the present findings in that perspective-taking abilities likely influence perceived expectations of cooperation and reciprocity, rather than general generosity.

The current study used a single false-location task as the measure of theory of mind abilities [19]. It is not the contention herein that a single measure of false-belief is entirely encompassing of perspective-taking abilities [27], yet the task does differentiate between children with very rudimentary early perspective-taking and those without. Future studies may benefit from a more scaled measurement of theory of mind, enabling finer-grained examinations of the developing relation between perspective-taking and generosity. It is also possible that children who fail a false-belief task have lower verbal abilities and may have misunderstood the directions of the dictator game task, yielding greater sharing with another individual. However, in the present study children’s understanding of the dictator game directions was subject to several rule checks wherein the experimenter would guide the child through the directions again if they failed any understanding “check,” greatly reducing the likelihood that greater sharing in children who fail false-belief was systematically due to misunderstanding of the dictator game. Additionally, other investigations using a children’s version of the dictator game have shown that preschool children have an impressive grasp of the dictator game [8,17], making this alternative interpretation less plausible.

Interestingly, these data also fail to show a relation between any aspects of developing executive function and expressive sharing behavior. While this lack of relation may be due to the types of assessments of EF used, it is important to note that other studies have also failed to show such a relation [10]. Furthermore, in the single study in which a relation was found, the relation was no longer significant when only including children who had shared any resources [14]. Taken together, the role of developing executive function in increased expressions of generosity across middle childhood begins to be questionable.

Overall, the development of generosity in children and its relation to general cognitive capacities, such as theory of mind and executive function, remains a topic of great interest for early social and moral behavior, and clearly warrants further investigation.

## Supporting Information

S1 Dataset (SAV)
